# Intimate Partner Violence in Pregnancy: Knowledge and Experiences of Pregnant Women and Controlling Behavior of Male Partners in Sokoto, Northwest Nigeria

**DOI:** 10.1155/2020/7626741

**Published:** 2020-03-06

**Authors:** Oche Mansur Oche, Habibullah Adamu, Aisha Abubakar, Munira Sahabi Aliyu, Abubakar Shehu Dogondaji

**Affiliations:** Department of Community Health, Usmanu Danfodiyo University, Sokoto, Nigeria

## Abstract

**Background:**

Violence against women perpetrated by an intimate partner is an important public health issue. In recent years, attention has focused also on intimate partner violence (IPV) during pregnancy due to its prevalence, adverse health consequences, and intervention potentials.

**Aim:**

To determine the knowledge, experiences, and factors influencing IPV, including the controlling behaviors of male partners of pregnant women attending an antenatal clinic (ANC) of a tertiary health facility in Sokoto. *Materials and method*. A descriptive cross-sectional study was conducted among 260 pregnant women attending ANC in a tertiary health facility in the Sokoto metropolis. They were selected using a systematic sampling technique, and a set of pretested questionnaire items was used for data collection. Data were analysed using IBM SPSS version 20.

**Results:**

The respondents' ages ranged from 19 to 40 years with a mean of 29.09 ± 4.99 years, and up to 83.5% of them were in a monogamous setting. Three-quarters of them were Muslims mostly from urban areas (72.1%), and 36.4% had a university or HND degree. Majority of them responded correctly to questions on IPV; overall, up to 99.2% of them had good knowledge of IPV. About 33% of the respondents have experienced IPV while pregnant and up to 61.7% of them said they did nothing because of fear. Some of the controlling behaviors of male partners included always asking for permission before seeing friends and family members and also controlling their finances. Factors associated with IPV include tribe, place of residence, and partner consuming alcohol.

**Conclusion:**

Majority of the respondents had good knowledge of IPV with about one-third of them ever experiencing it. Respondent's partners were mostly jealous and exhibited some form of controlling behaviors. Physical violence was the most prevalent form, and most of the victims did nothing about it. Government and women's right groups should push for the implementation of tougher punitive measures against perpetrators of IPV.

## 1. Introduction

Violence against women is a major public health and human rights concern, with intimate partner violence and sexual violence being among the most pervasive forms of violence against women [1]. Although women can be violent in relationships with men, the most common perpetrators of violence against women are male intimate partners or ex-partners [2]. Until recently, most governments have considered violence against women (particularly “domestic” violence by a husband or other intimate partner) to be a relatively minor social problem [3]. In recent times, however, violence against women is recognized as a global concern [3, 4]. Intimate partner violence (IPV) is defined as threatened, attempted, or completed physical or sexual violence or emotional abuse by a current or former intimate partner. It describes the physical, sexual, or psychological harm by a current or former intimate partner or spouse, and this type of violence can also occur among heterosexual or same-sex couples [4]. The World Health Organization (WHO) defines intimate partner violence as an act of coercion, physical abuse, or threat of violence in an intimate relationship [5].

According to WHO, IPV is the most common form of violence against women. Violence by an intimate partner is manifested by physical, sexual, or emotional abusive acts as well as controlling behaviors; although violence occurs in different forms and settings including the workplace, school, and community, violence at home by intimate partner violence is considered as the most prevalent form [6]. The act of physical violence includes slapping, kicking, pushing, and beating, as well as forced sexual intercourse and other forms of sexual coercion. Psychological abuse involves insults, belittling, constant humiliation, threats of harm, or controlling behaviors that consist of isolating a person from friends and families; monitoring their movements; and restricting access to financial resources, employment, education, or medical care [7].

Studies conducted in sub-Saharan African and Asian countries showed an IPV rate ranging from 28% in Madagascar, 74% in Ethiopia, and 57% in India to 87% in Jordan [8]. In a multicountry study conducted in 10 different countries, a rate ranging from 18.5 to 75.8% was reported; domestic violence by an intimate partner alone had a rate of 15.5 to 70.9%, while violence by nonpartners ranged between 5.1 and 64.6% [5]. In Nigeria, a study conducted in Lagos, Southwest Nigeria, on the prevalence and predictors of intimate partner violence exposure showed a one-year prevalence of 29%, with significant proportions reporting psychological (23%), physical (9%), and sexual (8%) abuse, while in Oyo, a study showed that there was a 31.1% prevalence of wife beating among women of reproductive age [9, 10]. In Northern Nigeria, studies conducted among pregnant women in Zaria and Jos showed 28% and 63.2% of the respondents, respectively, experienced some form of abuse [11, 12].

Intimate partner violence in pregnancy has been identified among the leading causes of maternal mortality in some developed countries like the United States and the United Kingdom [13]. Pregnancy-related IPV has been reported to be associated with high perinatal and neonatal mortality risk among exposed women compared to unexposed pregnant women [14]. Neonatal complications include intrauterine growth retardation, preterm delivery, and low birth weight with extended intensive hospitalization [15–19].

Maternal consequences associated with IPV during pregnancy include abortions, miscarriages, preeclampsia, gestational diabetes, and placental abruption [20].

Although the prevalence of IPV is quite high in Nigeria, far fewer cases are reported. This is probably because of the influence of religion and culture especially in many parts of Africa, where culture may allow couples to solve their problem by the use of violence, since most cases of violence against an intimate partner are not seen as wrong. Nigeria still remains patriarchal in nature, where men are regarded as “gods” of the household, controlling every affair, including the women's right to reproductive capabilities [21]. Incidents are therefore, underreported because doing so is viewed as causing indignity to the husband and being disrespectful of family members and elders whose roles include arbitrating in such matters. As a result of this, the true magnitude of the problem is relatively unknown and unexamined [22–24].

Despite increasing research on the prevalence and health effects of IPV during pregnancy from numerous countries around the globe, several gaps in knowledge still exist especially in low- and middle-income countries including Nigeria [25]. Though several studies have been conducted on IPV globally, in Nigeria there is still dearth of information on IPV; most of the studies conducted looked at IPV among women generally, but not much studies had been carried out among pregnant women in Sokoto State despite its effect on the health of the mothers and their babies. Systematic reviews were conducted on domestic violence, which included studies done in different parts of the world; however, studies among pregnant women were not included in the review. The findings showed that relatively few studies and publications emerged from Africa compared to North America and Europe [26]. Furthermore, there are differences in cultural and religious patterns in the different zones in the country; even in the northern part of the country, there are differences in what people regard as IPV [27].

This study, therefore, is aimed at examining the knowledge of IPV, controlling behaviors of male partners, and experiences of intimate partner violence among women attending an antenatal clinic at the Usmanu Danfodiyo University Teaching Hospital, Sokoto.

## 2. Materials and Method

The study was conducted at the Usmanu Danfodiyo University Teaching Hospital (UDUTH), Sokoto between June and August, 2018. Being a tertiary institution, the hospital provides specialized health care service to Sokoto State, the entire northwestern region of the country, and the neighboring Niger Republic. With a bed capacity of 850, UDUTH has staff strengths of over 1705, which includes doctors, nurses, pharmacists, medical laboratory scientists, and physiotherapists spread across all departments providing curative, preventive, and rehabilitative services. Antenatal clinic service is provided on all the weekdays with an average daily attendance of 250 pregnant women.

The study employed a descriptive cross-sectional study design, and all pregnant women presenting at the ANC clinic for booking or routine antenatal care and must have had a previous pregnancy (inclusion criteria) constituted the study population. Respondents were recruited into the study using the formula for estimating sample size in a population less than 10,000 [28].

After adjusting for a nonresponse rate of 10%, a total of 260 respondents were recruited into the study.

A systematic sampling technique was used to select the study participants after calculating the sampling interval as follows:

(1) $$\begin{array}{c} \text{Sampling interval }\left(k\right)=\text{number of pregnant women per booking clinic},\\ \text{Sample size}=\frac{100}{264},\\ K=\frac{1}{3}. \end{array}$$

Based on the above sampling interval, the systematic sampling technique was carried out as follows:

- The first participant was selected using simple random sampling carried out among the first three pregnant women that came for booking
- Thereafter, every third pregnant woman that came to the ANC clinic for booking was enrolled in the study until the required sample size was obtained. This was continued every day until the desired sample size was obtained

A set of pretested semistructured interviewer-administered questionnaire items was administered on the respondents which sought information on respondents' sociodemographic characteristics, their knowledge of IPV, the controlling behavior of partners during pregnancy, the experiences of respondents, and factors influencing IPV during pregnancy.

Data collection using the instrument described above was done with the help of three medical students who were trained by the researchers on the objectives of the study, general principles of research ethics, interpersonal communication, and techniques of data collection.

The data from the questionnaire was manually checked for completeness and entered into IBM SPSS version 20 for electronic data cleaning and analysis. Each correct response to a knowledge variable was awarded a score of one mark, and a zero mark was awarded to each incorrect response. The knowledge scores were added up, converted to percentage, and graded as either good knowledge (score of ≥50%) or poor knowledge (<50%). Continuous variables were summarized as mean and standard deviation, and categorical variables were summarized and presented as frequencies and percentages. This was followed by inferential statistics (bivariate analysis), which were used to identify the major determinants of IPV during pregnancy. The level of statistical significance was set at 5% (*p* < 0.05).

Permission for the study was obtained from the ethics and research committee of UDUTH. Participants were informed of the objectives of the study and were assured of the confidentiality of the information volunteered. Informed verbal consent was also obtained from all the respondents.

## 3. Results

The respondents' ages ranged from 19 to 40 years with a mean age of 29.09 ± 4.99 years, and up to 83.5% of them were in a monogamous setting. Up to three-quarters of them were Muslims mostly from urban areas (190 (72.1%)), and 92 (36.4%) had a university or HND and 96 (36.9%) belonged to the upper socioeconomic class (SEC); for their partners, 170 (65.4%) were in the upper SEC (Table 1).

**Figure 1:**
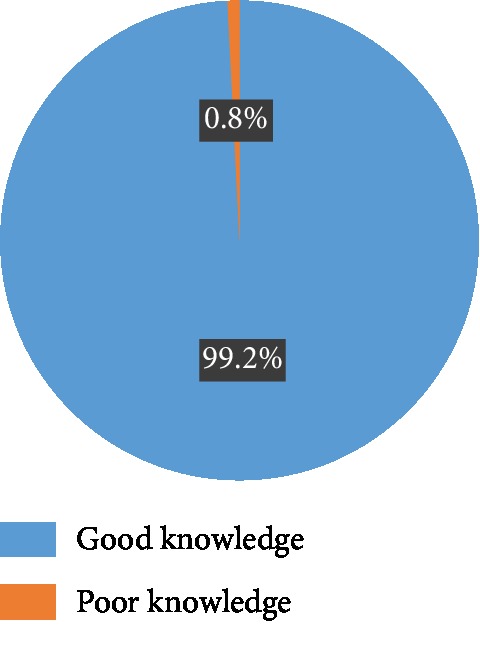
Knowledge of intimate partner violence.

Majority of the respondents (239 (92.3%)) were aware of what intimate partner violence is, and a greater proportion of them (102 (39.2%)) heard it from the media. Majority of them responded correctly to questions on IPV; overall, up to 258 (99.2%) of them had good knowledge of IPV (Table 2 and Figure 1).

**Figure 2:**
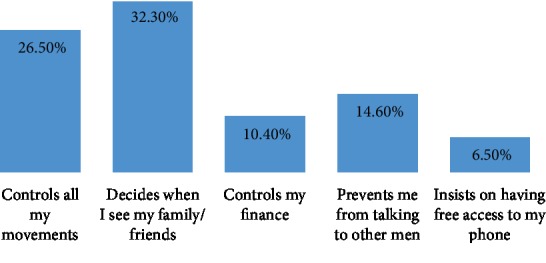
Controlling behavior of partner (multiple responses considered).

About one-third of the respondents (84 (32.3%)) said they have to seek for their partners' permission before seeing friends and family, and partners controlled and monitored all their movements (69 (26.5%)) and controlled their finances (28 (10.6%)) (Figure 2).

**Figure 3:**
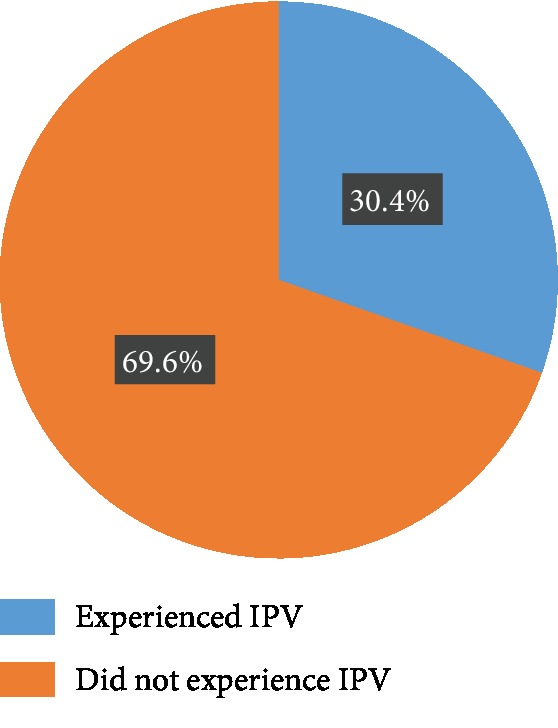
Lifetime prevalence of IPV during pregnancy.

**Figure 4:**
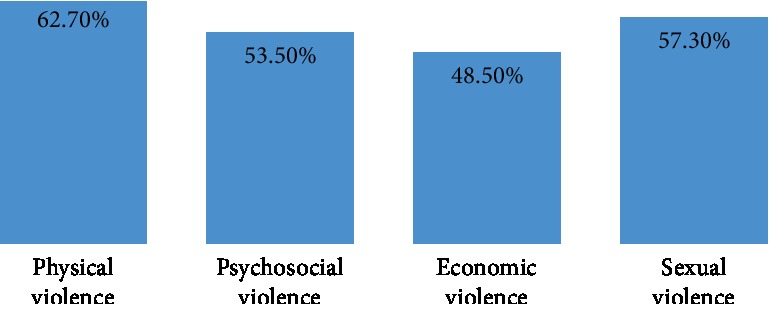
Forms of IPV in pregnancy experienced by respondents (multiple responses considered).

The lifetime prevalence of IPV in pregnancy was 30.4% (*N* = 79) (Figure 3); a total of 26 (32.9%) of the women experienced IPV in their first pregnancies, while 53 (67.1%) of them occurred during their subsequent pregnancies. About two-thirds of the respondents said the IPV they experienced occurred frequently, and 44 (55.7%) of them said it was less frequent during pregnancy than outside pregnancy. The most common forms of IPV were physical and sexual violence (62.70 and 57.30%, respectively (Figure 4)). Other forms of IPV experienced by respondents include being insulted (78 (30%)), being humiliated (61 (23.5%)), and being forced to have sexual intercourse (39 (15%)) (Table 3).

Regarding the reactions of respondents following the incidences, up to 46 (61.3%) of them admitted doing nothing following IPV, mainly because of fear (35 (77.8%)). Majority of the respondents (236 (90.8%)) believed that IPV is associated with drugs and alcohol consumption, while 249 (95.8%) of them were of the opinion that it is associated with exposure to violence between parents (Table 4).

Regarding factors associated with IPV during pregnancy, up to 31 (62.0%) of those who are of the Yoruba tribe had experienced IPV, whereas only 25 (14.8%) among the Hausa tribe experienced it; the relationship was statistically significant (*X*^2^ = 71.280, *p* < 0.001). Similarly, more than half (39 (55.7%)) of those who lived in rural areas experienced IPV as against those living in urban areas and the relationship was statistically significant (*p* < 0.001). Other factors significantly associated with IPV in pregnancy were age (*p* = 0.008), religion (*p* < 0.001), SEC class of both partners, witnessing IPV during childhood (*p* < 0.001), and consumption of alcohol and illicit substances by both partners (*p* < 0.001). Factors such as length of relationship, parity, and knowledge of IPV were not significantly associated with IPV (Table 5).

## 4. Discussion

Intimate partner violence is a reality that affects people in all walks of life and has remained a problem of public health importance. In this study, the mean age of the respondents was 29.09 ± 4.99 years, which is similar to what was reported in a study on workplace violence and sexual harassment in Ethiopia [29]. The similarity observed in both studies could be attributed to the fact that more than half of the respondents were below 30 years of age; moreover, up to 47.3% of them were within the age group of 20-29 years. More than two-thirds of the respondents in this study were Hausa Muslims, and this could be a reflection of the study area which predominantly comprises of Muslims and people of Hausa ethnicity. However, there is also a notably significant proportion of other tribes including the Yoruba and Igbo and this is understandable as this study was conducted in a urban area which is mainly cosmopolitan.

Those who had completed their tertiary education constituted the highest proportion of the respondents (96 (36.4%)), and this is probably because the study was conducted in a teaching hospital located within the metropolis; moreover, most people who belong to the high echelon of society preferred sending their wives to the teaching hospital for ANC where we have an array of specialist gynecologists. The findings were comparable to studies carried out within and outside of Nigeria [2, 11, 30].

Majority of the respondents (92.3%) were aware of what IPV is, and a greater proportion of them 102 (39.2%) heard about it from the media. This high level of awareness observed is not surprising because this study also revealed that up to 99% of the respondents had good knowledge of IPV. Studies conducted in Delta State and Abuja, Nigeria, also made similar observations [31, 32].

Most of the respondents had correct responses to the question regarding forms of violence; however, questions relating to physical violence had more correct responses compared to questions on psychological and sexual violence. The high level of knowledge of the physical form of IPV observed in this study and other studies may likely be attributed to the influence of culture on the perception of what constitutes violence, such that most women especially in Africa only consider the physical form of IPV as violence but do not consider some other forms of IPV as violence. As a result of cultural factors, women tend to believe once you are married, it is the man's right to demand for sex at any time and forcing his spouse to have sexual intercourse is not considered as sexual violence; even the Nigerian law does not clearly state anything regarding issues like marital rape [33–35]. Studies in rural Ethiopia and America on cultural difference in knowledge of violence among Hispanics, African American, and Polish residents also made similar observations where most of the women considered only physical violence as IPV [36, 37]. This underscores the need to educate women on other forms of IPV in a culturally acceptable manner. In terms of overall knowledge of IPV, this study observed that up to 99% of the respondents had good knowledge of IPV and this is much higher than the 72% and 75.7% reported in studies conducted in Jos and Kano Nigeria, respectively [38, 39]. This high proportion of respondents with good knowledge of IPV is not surprising because more than half of the respondents had up to tertiary level education.

Controlling behaviors reported by respondents include having to always ask for permission before seeing friends and family (32.6%) and partners controlling all their movements, including controlling their finances (10.6%). These findings are similar to what was reported in a study involving the use of secondary data from NDHS 2008 but lower than the 43.3% reported in another study conducted in Kano [27, 40]. Controlling behavior by the husband/partner has been shown to be associated with both physical and sexual IPV [41, 42], and it is a reflection of the increased vulnerability to abuse by women residents in societies that validate a male-dominated family structure and social order and encourage men to exercise control over women. This finding is in support of the feminist theory [43] and is also in favor of the hypothesis that controlling behavior is associated with increased likelihood of violence, most likely acting as a precursor to violence.

Close to one-third of the respondents had experienced IPV at least in one of their pregnancies, with a significant proportion (65.8%) of women reporting frequent exposure by their husband/male partner. This is in tandem with the finding from the study by Sigalla et al. in Tanzania where a prevalence of IPV in pregnancy of 30% was observed [44].

However, the prevalence of IPV recorded in our study is lower than what was reported from similar studies on magnitude and correlates of IPV in Ethiopia [45] and in Southwest Nigeria [46], where the lifetime prevalence of intimate partner violence was 70%. The lower prevalence in this study could probably be due to the fact that this study looked at IPV in pregnancy as against the other studies that looked at lifetime prevalence both in and outside pregnancy. Despite the fact that the prevalence of IPV observed in this study is lower than the lifetime prevalence observed in other studies, it still carries huge public health implications because IPV in pregnancy has been shown to be associated with a higher rate of maternal and foetal outcomes [19, 47–50].

Of the various forms of IPV experienced during pregnancy, physical violence was the most common (62.7%). Studies conducted in Kano and Oyo States reported lower prevalences [40, 51]. However, higher rates of physical violence were also reported in studies conducted in southwest Ethiopia, Tanzania, eastern Nigeria, Bangladesh, Ukraine, and Peru [45, 50–54].

Close to two-thirds of the study subjects opined that they received kicks on the abdomen, beating, and choking whereas a smaller proportion (17.8%) said pushing, shoving, pulling of hair, and slapping were the forms of physical violence that they experienced. Empirical evidence has shown that such kicks to the pregnant abdomen do result in devastating effects on both the mother and the foetus; in a Tanzanian study, 23 and 38% of all women exposed to physical violence during pregnancy suffered blows to the abdomen [50] which may lead to placental damage, rupture of the membrane, and consequently premature uterine contractions [55].

These forms of physical violence were also reported from other studies within and outside Nigeria; for example, in a study conducted in Southwest Nigeria, the major types of physical violence experienced were being slapped (27.2%), being kicked (14.4%), and being hit (13.4%), [56] and in Malawi 20% reported being pushed, shaken, slapped, or punched [57]. A little above half (57.3%) of the respondents experienced sexual violence during pregnancy. A World Health Organization multicountry study observed that the lifetime prevalence of sexual violence by an intimate partner in most sites studied was between 10 and 50% [5]. Sexual violence was reportedly lower compared to physical violence probably because of the fact that issues of sex are still regarded as a taboo and should not be openly discussed [35, 36]. In a similar study carried out in Lima, Peru, the lifetime prevalence of sexual violence was 8.7% [58].

Regarding the action taken by respondents after exposure to IPV, up to 61.3% of them said they did nothing and the reason given by 77.8% of them was that it was because of fear of repercussions. In a study on the disclosure of IPV conducted in Lagos, slightly more than half (54%) of the respondents agreed that they would not disclose their experiences of violence to anyone [59]. Traditionally, women especially in rural areas are often encouraged to stay in abusive relationships without disclosing their experiences to anyone, due to the strong cultural belief that a woman's place is with her husband and also because divorced and separated women are not held in high social esteem compared to women who remain in marriage. This could well be the reasons why most felt reporting was unnecessary and couples should rather handle their own issues as culture and religion advocates that women should learn to endure and be patient in all circumstances. Anecdotal evidence in most parts of Nigeria showed that mothers of victims of IPV would ask them to remain in their husbands' homes saying “after all, we the mothers went through the same experiences in the hands of your fathers”.

Regarding factors associated with IPV during pregnancy, this study revealed that the tribe of the respondents was significantly associated with IPV; up to 62% of those who are of the Yoruba tribe had experienced IPV, whereas only 14.8% from among the Hausa tribe experienced it. This is not surprising because according to the NDHS 2013 report, IPV in pregnancy is higher in Southwest Nigeria (where Yoruba is the predominant tribe) than in Northwest Nigeria which has the highest proportion of people of the Hausa tribe in the country [27]. This may not be related to the fact that the sociocultural milieu of Sokoto State, the study area, does not encourage the reporting of incidents of IPV. Also, more than half (55.7%) of those who lived in the rural areas experienced IPV compared to those living in urban areas and this is similar to what was observed in other studies from Southwest Nigeria [23, 46].. However, findings from a study in Southwest Nigeria and Ethiopia were at variance with what was obtained in this study, where those with formal education formed a larger proportion of those experiencing abuse [45, 59]. This variation shows that violence cuts across all groups, and the belief that only women who are uneducated face violence may be exaggerated because there are many other factors that contribute to women's risk of intimate partner violence. Other factors significantly associated with IPV in pregnancy are age, religion, witnessing IPV during childhood, and consumption of alcohol and illicit substances by both partners. Evidence has shown that IPV occurred more among those whose partners consumed alcohol [51]. This suggests that if alcohol consumption by male intimate partners can be well controlled, then the prevalence of IPV may also be reduced significantly. Factors such as length of relationship, parity, and knowledge of IPV were not significantly associated with IPV.

Findings from this study buttresses the fact that the occurrence of IPV is an interplay of different factors which may solely be due to differences in individuals, culture, and the society, as what is obtained in one setting, even though it may be similar, may not apply in another.

## 5. Conclusion

A very high proportion of the respondents had good knowledge of IPV, and about one-third of them had experienced IPV in pregnancy. A sizeable number of the respondents that experienced IPV did nothing about it, mainly due to fear of what might follow. This fear may not be unrelated to the sociocultural milieu of the study area where religion controls the social lives of the inhabitants especially the womenfolk. Factors associated with IPV include tribe, place of residence, level of education of partners, and alcohol consumption. Increased public awareness on the dangers inherent in IPV should be intensified while governments at all levels and law enforcement agencies should ensure that the perpetrators are made to face the law and stiffer penalties are meted out to perpetrators of IPV, while at the same time encouraging victims of IPV to speak out regardless of length of onset. Women's organizations must be in the vanguard for the fight against IPV and to come up with avenues and measures to break the cycle of silence and to fight for the rights of women, while also rehabilitating victims of such acts.

### 5.1. Study Limitations

Intimate partner violence is a culturally sensitive issue in this part of the world and victims are afraid of retributions, hence some may inadvertently hoard important information. We tried from the onset to allay their fears and assure them of the confidentiality of any information divulged to the researchers.

## Figures and Tables

**Table 1:** Sociodemographic characteristics of respondents.

Variables	Frequency (%)
Age group (years)	
<20	13 (5.0)
20-29	123 (47.3)
30-39	122 (46.9)
40-49	2 (0.8)
Mean	29.09 ± 4.99
Marital status	
Married	259 (99.6)
Widowed	1 (0.4)
Type of marriage	
Monogamous	217 (83.5)
Polygamous	43 (16.5)
Religion	
Islam	198 (76.2)
Christianity	61 (23.5)
Others	1 (0.4)
Tribe	
Hausa	169 (65.0)
Yoruba	50 (19.2)
Igbo	21 (8.1)
Fulani	20 (7.7)
Place of residence	
Urban	190 (72.1)
Rural	70 (26.9)
Level of education of wife	
University/HND graduate	92 (36.4)
Diploma/NCE/SSCE	34 (13.1)
Completed primary school/JSS	47 (18.1)
Primary school not completed/	13 (5.0)
Qur'anic school only/none	74 (28.5)
Level of education of husband or partner	
University/HND graduate	140 (53.8)
Diploma/NCE/SSCE	55 (21.2)
Completed primary school/JSS	49 (18.8)
Primary school not completed/	6 (2.3)
Qur'anic school only/none	10 (3.8)
Occupation of wife	
Senior civil servant/professional/manager/contractor/large scale business	63 (24.2)
Intermediate school civil servant/secondary school teacher	14 (5.4)
Junior secondary school teacher/driver/artisan	53 (20.4)
Petty trader/labourer/messenger	12 (4.6)
Subsistent farmer/student/full-term house wife	118 (45.4)
Occupation of husband/partner	
Senior civil servant/professional/manager/contractor/large scale business	133 (51.5)
Intermediate school civil servant/secondary school teacher	39 (15)
Junior secondary school teacher/driver/artisan	42 (16.2)
Petty trader/labourer/messenger	38 (14.6)
Subsistent farmer/student/full-term house wife	7 (2.7)
Wife's SEC	
Upper class	96 (36.9)
Middle class	41 (15.8)
Lower class	123 (47.3)
Husband/partner's SEC	
Upper class	170 (65.4)
Middle class	53 (20.4)
Lower class	37 (14.2)

**Table 2:** Knowledge of IPV among pregnant women.

Variables	Frequency (%)
Have you heard of IPV?	
Yes	239 (92.3%)
No	20 (7.7)
Source of information on IPV	
Media	102 (39.2%)
Personal experience	34 (13.1%)
Hospital/health worker	1 (0.4%)
Lecture/seminar	104 (40%)
IPV is a serious public health issue	
Yes	253 (97.3%)
No	7 (2.7%)
Slapping, kicking dragging, beating, choking, pushing, etc., are examples of IPV	
Yes	250 (96.2%)
No	10 (3.8)
Forcing partner to have sex when he/she does not want to	
Yes	227 (87.3%)
No	33 (12.7%)
Forcing partner to do something sexual that he/she finds degrading or humiliating	
Yes	219 (84.2%)
No	41 (15.8%)
Belittling or humiliating a partner in front of other people	
Yes	230 (88.5%)
No	30 (11.5%)
Restricting a partner from contact with family or friends	
Yes	243 (93.5%)
No	17 (6.5%)
Denial of food and other nutritional substance	
Yes	231 (88.8%)
No	29 (11.2)

**Table 3:** Experience of women on various forms of IPV during pregnancy.

Variables	Frequency (%)
Have you ever experienced IPV during pregnancy?	
Yes	79 (30.4%)
No	181 (69.6%)
If yes, during which pregnancy was IPV experienced?	
First	26 (32.9%)
Subsequent pregnancies	53 (67.1%)
Was the pregnancy you experienced IPV desired and planned for?	
Yes	217 (83.5%)
No	43 (16.5%)
Number of times you ever experienced IPV during pregnancy?	
Once	13 (16.5%)
Twice	9 (11.4%)
More than twice	5 (6.3%)
Frequently	52 (65.8%)
Magnitude of intimate partner violence during pregnancy compared to outside pregnancy	
More frequent	7 (8.8%)
Less	44 (55.7%)
Same	28 (35.4%)
Type of physical violence experienced during pregnancy	
Pushing, shoving, pulling hair, or slapping	14 (17.8%)
Kick on the abdomen, beating, or choking	58 (73.4%)
Thrown something at, attempted burning	7 (8.8%)
Has your partner ever insulted or made you feel bad?	
Yes	78 (30%)
No	182 (70%)
Did your partner ever belittle or humiliate you before people	
Yes	61(23.5%)
No	199 (76.5%)
Has your partner/husband ever taken your earnings or savings against your will?	
Yes	48 (18.5%)
No	212 (81.5%)
Has your partner ever refused to give you money for household expenses?	
Yes	78 (30%)
No	182 (70%)
Have you ever been forced by your partner to have sexual intercourse by threatening you or withholding certain things?	
Yes	39 (15%)
No	221 (85%)
Do you agree to have sexual intercourse with your partner when you do not want to?	
Yes	110 (42.3%)
No	150 (57.7%)

**Table 4:** Reactions of respondents following their experience of IPV during pregnancy.

Variables	Frequency (%)
If you have experienced IPV during pregnancy, what did you do?	
Nothing	46 (61.3%)
Reported him to relations	19 (25.3%)
Quit relationship	10 (13.3%)
If nothing, why?	
Fear	35 (77.8%)
I forgave him	10 (22.2%)
Did you seek for medical care following the effect of the violence?	
Yes	58 (79.5%)
No	15 (20.5%)
If yes, how were you managed?	
In-patient	29 (50.9%)
Out-patient	28 (49.1%)
If you did not seek for medical care why?	
Due to fear	5 (33.3%)
Lack of finance	3 (20.0%)
Shame	7 (46.7%)
If you have experienced IPV during pregnancy, did anyone try to help you out?	
Yes	40 (51.9%)
No	37 (48.1%)
If yes, who?	
Family	24 (60.0%)
Close friends	16 (40.0%)
IPV is associated with harmful use of drugs and alcohol	
Yes	236 (90.8%)
No	24 (9.2%)
IPV is associated with exposure to violence between parents	
Yes	249 (95.8%)
No	11 (4.2%)
IPV affects the victim's health status and lifestyle	
Yes	250 (96.2%)
No	10 (3.8%)
Do you think IPV during pregnancy should be encouraged by the society?	
Yes	0 (0.0%)
No	260 (100%)

**Table 5:** Factors associated with IPV during pregnancy.

Variables	Ever experienced IPV during pregnancy?		Test statistic
	Yes	No	
Age (years)			
<20 years	0 (0)	13 (100)	*X* ^2^ = 11.105
20-29 years	47 (38.2)	76 (61.8)	*p* = 0.008
30-39 years	32 (26.2)	90 (73.8)	
40-49 years	0 (0)	2 (100)	
Tribe			
Hausa	25 (14.8)	144 (85.2)	*X* ^2^ = 71.286
Yoruba	31 (62.0)	19 (38.0)	*p* < 0.001
Igbo	6 (28.6)	15 (71.4)	
Fulani	17 (85.0)	3 (15.0)	
Religion			
Islam	39 (19.7)	159 (80.3)	*X* ^2^ = 45.44
Christianity	39 (63.9)	22 (36.1)	*p* < 0.001
Others	1 (100)	0 (0)	
Place of residence			
Urban	40 (21.1)	150 (78.9)	*X* ^2^ = 29.055
Rural	39 (55.7)	31 (44.3)	*p* < 0.001
Wife's SEC			
Upper class	5 (5.2)	91 (94.8)	*X* ^2^ = 47.49
Middle class	15 (36.6)	26 (63.4)	*p* < 0.001
Lower class	59 (48.0)	64 (52.0)	
Husband/partner's SEC			
Upper class	36 (21.2)	134 (78.8)	*X* ^2^ = 19.709
Middle class	25 (47.2)	28 (52.8)	*p* < 0.001
Lower class	18 (48.6)	19 (51.4)	
Parity			
Primi	5 (14.3)	30 (85.7)	*X* ^2^ = 5.054
Multipara	58 (32.4)	121 (67.6)	*p* = 0.076
Grand multipara	16 (34.8)	30 (65.2)	
Length of relationship			
1-5 years	19 (26.4)	53 (73.6)	*X* ^2^ = 3.820
6-10 years	46 (35.9)	82 (64.1)	*p* = 0.149
>10 years	14 (23.3)	46 (76.7)	
Witnessed IPV during your childhood?			
Yes	30 (88.2)	4 (11.2)	*X* ^2^ = 61.887
No	49 (21.7)	177 (78.3)	*p* < 0.001
Who chose your partner for you?			
Parents	16 (34.8)	30 (65.2)	*X* ^2^ = 0.511
Myself	63 (29.4)	151 (70.6)	*p* = 0.483
Who takes the most important decision for your family?			
My partner	16 (100)	0 (0)	*X* ^2^ = 52.342
Both of us	58 (24.3)	181 (75.7)	*p* < 0.001
In-laws	5 (100)	0 (0)	
Do you consume alcohol?			
Yes	8 (88.9)	1 (11.1)	*X* ^2^ = 15.085
No	71 (28.3)	180 (71.7)	*p* < 0.001
Does your partner consume alcohol?			
Yes	41 (95.3)	2 (4.7)	*X* ^2^ = 102.795
No	38 (17.5)	179 (82.5)	*p* < 0.001
Do you use illicit drugs/substances?			
Yes	29 (96.7)	1 (3.3)	*X* ^2^ = 71.403
No	49 (21.4)	180 (78.6)	*p* < 0.001
Does your partner use illicit drugs/substances?			
Yes	6 (85.7)	1 (14.3)	*X* ^2^ = 10.411
No	73 (28.9)	180 (71.1)	*p* = 0.004
Is your partner very jealous?			
Yes	75 (32.5)	156 (67.5)	*X* ^2^ = 3.894
No	4 (14.3)	24 (85.7)	*p* = 0.052
Knowledge of IPV			
Good knowledge	2 (100)	0 (0)	*p* = 0.091
Poor knowledge	76 (29.7)	180 (70.3)	(Exact)

## Data Availability

Data/results and narratives are in tandem with what the authors have inputed.
